# Study on the Thermodynamic Properties of Concrete Surface during Microwave Deicing of Airport Pavement

**DOI:** 10.3390/ma13163557

**Published:** 2020-08-12

**Authors:** Haowen Chen, Jinyu Xu, Yunquan Wu, Junliang Liu, He Huang

**Affiliations:** 1 Aeronautics Engineering College, Air Force Engineering University, Xi’an 710038, China; xujinyu169@163.com (J.X.); liujunliangafeu@163.com (J.L.); huangheafeu@163.com (H.H.); 2 College of Mechanics and Civil Architecture, Northwestern Polytechnical University, Xi’an 710021, China; 3 Air Force Guangzhou Engineering Design Office, Guangzhou 510050, China; wuyunquan9@163.com

**Keywords:** airport pavement, concrete, microwave deicing, thermodynamic properties, temperature distribution law

## Abstract

In the cold belt area, the icing phenomenon often appears on the airport pavement, which affects the safety of aircraft take-off and landing. Microwave deicing technology can effectively solve this practical problem and has many advantages. Taking microwave deicing technology as the research object and the surface temperature of pavement concrete as the index, an open microwave test system has been established in this paper. Based on this system, the temperature distribution, variation rule, and influencing factors of concrete have been studied systematically. The results show that the surface temperature of concrete increases linearly due to microwave action. In addition, during the microwave action, the temperature increase of the concrete surface is centered on the center point and shows a stepwise decreasing trend along the radius. At the same time, the increase of microwave source height leads to the increase of surface temperature distribution uniformity. The surface ice affects the rate of temperature increase of concrete in stages under microwave action, and the surface edge area remains frozen for a certain period of time. The temperature distribution of concrete surface is a decisive factor affecting the degree of ice removal, and the concrete surface will generate residual heat after the microwave action ends. This phenomenon can delay the regression of the temperature distribution of the concrete surface, thus effectively preventing the re-freezing of the ice layer.

## 1. Introduction

The airport pavement is a large-scale, open-faced, sheet structure that is exposed to the external environment for a long time. Therefore, climate conditions will have a greater impact on concrete pavement. The cold areas in northern China are characterized by wide distribution, heavy precipitation in winter and long duration of low temperatures [1]. This regional factor greatly affects the normal use of airport pavement during the winter period, thus threatening the safety and reliability of air transport. Therefore, fast and efficient deicing method is a hot issue that needs to be solved urgently at present. Traditional airport pavement deicing methods include mechanical cleaning method, deicing salt method, and hot blowing method. The hard and sharp ice-breaking device used in mechanical clearance aggravate the damage and aging of the airport pavement to a certain extent, which affects its structural strength, service performance, and durability [2,3]. Deicing salt is used to melt ice by lowering its freezing point. However, the long-term use of deicing salt may cause corrosion to the pavement and vehicles, and also pollute the surrounding soil and water environment [4,5]. Hot blowing is a kind of deicing operation using a jet deicing vehicle which is equipped with an aeroengine as the power unit. In practical operation, the high-temperature and high-speed air flow can easily damage the concrete structure and affect the thermal and mechanical properties of concrete under elevated temperatures [6,7,8,9].

As an environmentally friendly technology, microwave technology has developed rapidly in recent years. Mou et al. expounded the dielectric loss mechanism and the characteristics of microwave heating, explained the mechanism of microwave action on physical surface ice layer, and pointed out that this technology has broad development prospects [10]. Buchta and Wang et al. used Maxwell’s equation to derive the power density expression of the electromagnetic field energy loss in simple harmonic field, and analyzed the microwave heating principle [11,12]. Based on previous studies, Woodhouse discussed the physical properties of microwave and the polarization characteristics of electromagnetic wave and elaborated the interaction between microwave and matter [13]. In addition, microwave technology has also been applied to highway and airport pavement deicing in recent years and has achieved certain effects. By making absorbing concrete with taconite as the aggregate, Hopstock et al. proposed that incorporation of taconite could greatly increase the heating efficiency of road surface under the action of microwave [14,15]. Gallego used steel fiber as microwave absorbing component in asphalt concrete to increase the surface temperature of asphalt concrete, thereby improving deicing efficiency [16]. From the perspective of rapid deicing and optimal economy, Can et al. gave the best matching scheme of a new deicing design and put forward a standard evaluation method for deicing [17]. Gao tested the microwave heating performance of specimens with different types and contents of steel wool under the temperature of −5 and −10 °C [18]. By adding fly ash, carbonyl iron powder, iron oxide powder, and magnetite ore into cement concrete, Kang studied the effects of different materials and dosage on the mechanical properties of concrete and the efficiency of microwave deicing [19]. Liu took water–cement ratio, sand ratio, and graphite addition as variables to form pavement with different mixed properties, and then carried out experimental studies on dielectric properties, microwave deicing efficiency, and static mechanical properties of the specimen [20,21]. The above research results laid the foundation of microwave deicing technology on highway and airport pavement and achieved remarkable results. However, there are still blank areas not involved in the above research, which are as follows: firstly, in previous studies, asphalt concrete was the main research object. However, cement concrete and asphalt concrete have great differences in appearance and performance, resulting in different temperature distribution laws. Therefore, the existing conclusions are not completely applicable to microwave deicing of cement concrete. Secondly, the application objects of the above research were mostly highway pavement and were not applicable to airport pavement. Airport pavement concrete has the characteristics of large load bearing capacity and high frequency of use, which directly leads to the great difference in mechanical properties. However, there are relatively few systematic studies on microwave deicing of airport pavement concrete. Thirdly, the change of concrete surface temperature in microwave field directly reflects the efficiency of microwave deicing process. As the most intuitive characterization factor in deicing process, little research has been done on the temperature distribution of concrete surface. Therefore, the temperature distribution and its influencing factors of airport pavement concrete during microwave deicing process need to be studied urgently.

In view of the above problems, this paper takes the microwave deicing technology as the research subject, and develops a self-developed open microwave test system by taking the law of surface temperature variation of airport pavement concrete as the index. Based on this system, the temperature distribution law and its influencing factors of pavement concrete are studied systematically. The specific research contents include the influence of different microwave source height and ice layer on the surface temperature distribution of airport pavement concrete, ice layer breaking form, residual heat phenomenon, etc. This paper has certain significance to improve the microwave deicing method and further enhance the deicing efficiency.

## 2. Raw Materials and Specimen Preparation

### 2.1. Raw Materials

The test materials used in this paper were composed of ordinary Portland cement, gravel, sand, water, and water reducing agent. Among them, the cement is 42.5 ordinary Portland cement with fineness of 1.6. Concrete aggregates are limestone gravel with a density of 2.71 g/cm^3^, bulk density of 1.63 kg/L, and mud content of about 0.1%. Sand is river sand with a fineness modulus of 2.8 its density is 2.57 g/cm^3^ and its bulk density is 1.47 kg/L. Test water is drinking water. The water-reducing agent used is PCA type polycarboxylic acid water reducing agent with water reducing rate of 35%. Based on the mix design requirements of high-performance airport pavement and related research results [22], the basic mix design process of pavement concrete is as follows: firstly, the flexural strength of pavement concrete is calculated

(1) $$f_{cu,0}\geq f_{cu,k}+k\sigma$$

where $f_{cu,0}$ denotes the flexural strength of pavement concrete, $f_{cu,k}$ denotes the design strength of pavement concrete, *k* represents the assurance factor of concrete strength, and σ represents the flexural strength standard deviation of construction unit.

Then, according to the flexural strength of pavement concrete and the measured 28d strength of cement, the water cement ratio can be calculated by the equation

(2) $$f_{cu,0}=1.32f_{f}{}^{c}(0.96-W/C)$$

where $f_{cu,0}$ denotes the 28d flexural strength of pavement concrete, $f_{f}{}^{c}$ denotes the 28d flexural strength of cement, *W* and *C* represent unit water dosage and unit cement dosage in concrete respectively.

Finally, according to the absolute volume method, the sand ratio used in the mix ratio is calculated, and the final mix ratio of pavement concrete is obtained through trial mixing adjustment, as shown in Table 1 below.

### 2.2. Specimen Preparation

Mixing is one of the key steps in the preparation of concrete specimens, which determines the performance of concrete itself to a large extent, thus affecting the accuracy of test. In order to promote the formation of standardized specimen properties, this paper adopted the PC mixing method. The specific steps were as follows: first, mixed coarse aggregate and fine aggregate for 60 s, then poured in cement and continue to mix for 60 s, and finally added water and water reducing agent at the same time and mixed for 120 s.

**Figure 1:**
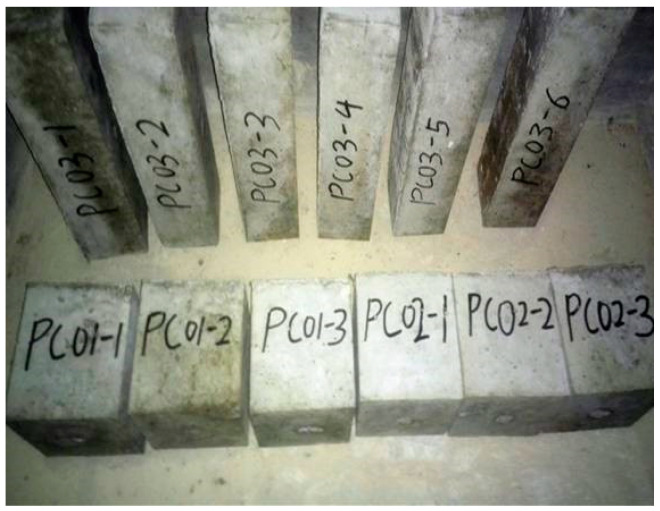
Concrete specimens.

After the concrete mixing process was completed, the concrete was poured into the mold of 150 × 150 × 150 mm^3^, and cured for 28 days under the standard state (T = 20 ± 2 °C, relative humidity > 95%). Subsequently, the concrete specimens were made, as shown in Figure 1.

After curing, we carried out the frozen ice layer treatment on the concrete surface. The specific methods were as follows: Rigid PVC board was used to surround and fix the specimen to ensure that the upper edge of the board was parallel to and 20 mm higher than the concrete edge, and the whole specimen was wrapped with plastic cloth. Subsequently, water was poured over the concrete surface, so that the water level just reached the upper edge of the PVC board but did not overflow. Finally, the specimen was placed in the low-temperature control cabinet for freezing.

**Figure 2:**
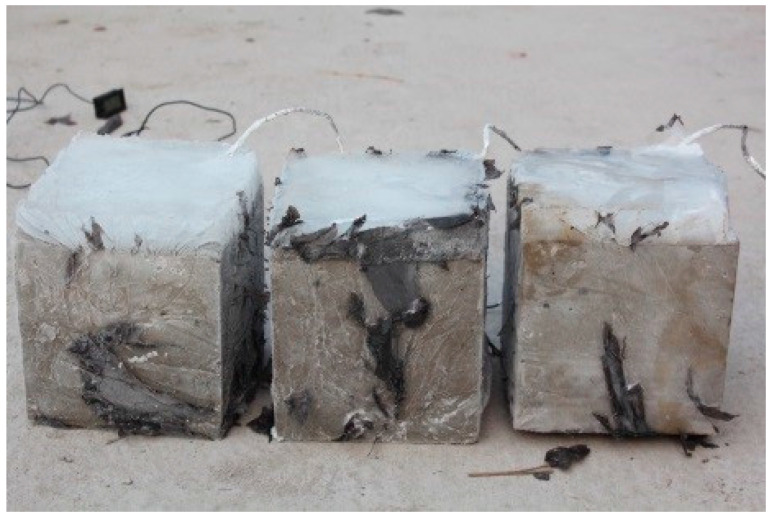
Concrete specimens of frozen ice.

After the completion of freezing, specimen could be prepared as shown in Figure 2 below. Due to the infiltration of some moisture, few ice crystals were frozen around the concrete specimen. Subsequently, the surrounding ice crystals were removed, and only the ice layer on the surface of the concrete specimen was tested.

## 3. Test Equipment and Test Method

### 3.1. Test Equipment

**Figure 3:**
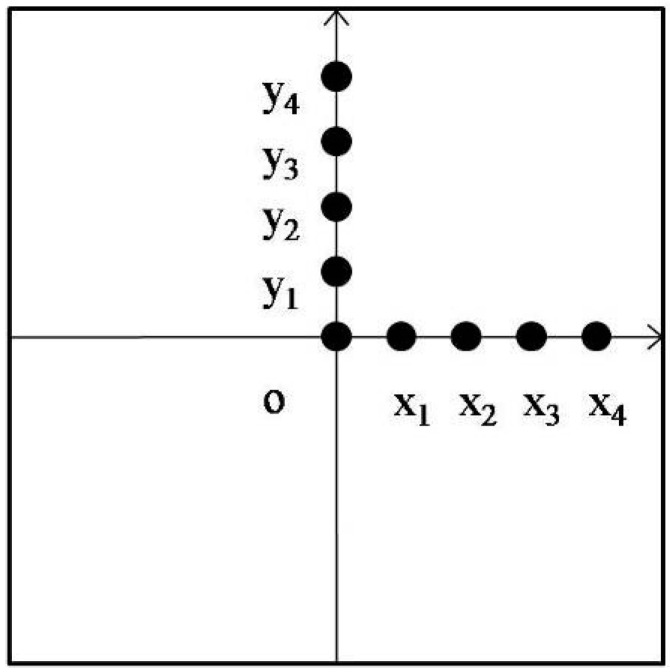
Schematic diagram of temperature sensor layout.

In order to measure the surface temperature of concrete directly, an infrared temperature measurement system was installed at the beginning of the design of the open microwave test system. However, due to limited space, only the center temperature and the edge temperature of the specimen were measured. In order to further explore the temperature distribution law, temperature sensors were used to collect data from more points. Since the port of the waveguide was rectangular and symmetrical in both left and right or upper and lower sides, the coordinate axis was established on the surface of the specimen with the center point as the coordinate origin. With the direction of the long side of the waveguide as the *x*-axis and the short side as the *y*-axis, a temperature measurement point was set every 15 mm in the positive direction of the *x*-axis and the *y*-axis. The central temperature measurement point was represented as o, and other temperature measurement points of *x*-axis and *y*-axis were represented as *x*_1_~*x*_4_ and *y*_1_~*y*_4_ respectively, as shown in Figure 3.

**Figure 4:**
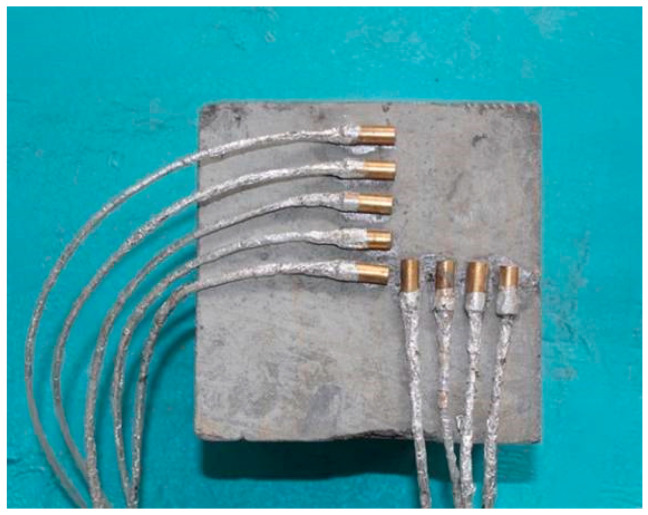
Surface state of specimen after placing the sensors.

Based on the above sensor placement method, temperature distribution of concrete surface was measured by embedded temperature sensor. The thermocouples used was WZP-128 thermal resistance (Xi’an, China), the temperature measurement range was from −30 to 250 °C, and the temperature measurement accuracy was 0.1 °C. Since sensors could not transmit signals well in the microwave field, the temperature sensor transmission line was further wrapped with tin foil paper so as to avoid interference of the microwave with the transmission line [23]. After the sensor was bonded and fixed with water glass, the surface state of the specimen could be shown in Figure 4.

**Figure 5:**
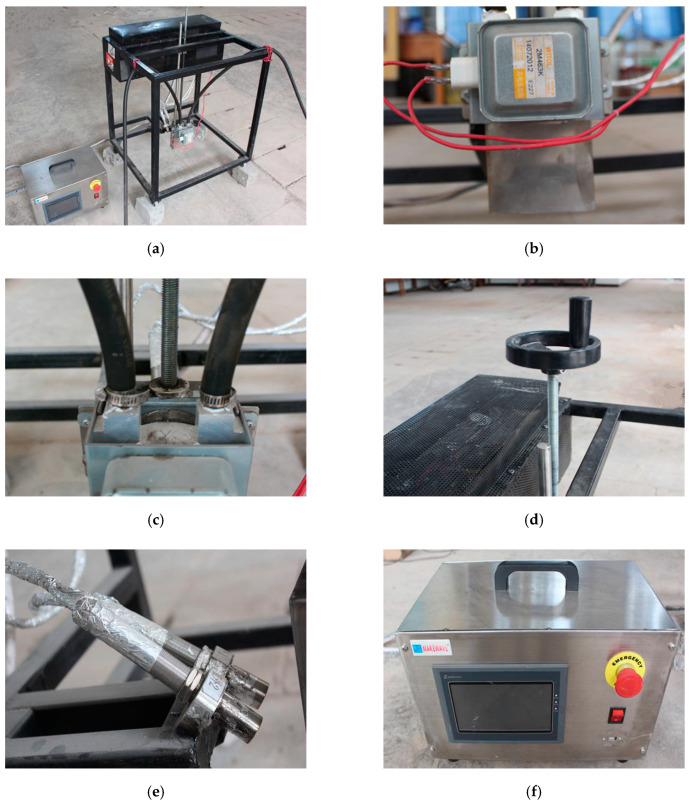
Microwave deicing test equipment: (**a**) Deicing main equipment; (**b**) Magnetron; (**c**) Water cooling pipeline; (**d**) Height control handle; (**e**) Infrared thermometer; (**f**) Remote control box.

In addition, the test equipment used for microwave deicing was an open microwave transmitting system designed independently. The equipment was composed of magnetron, water cooling device, height adjusting device, infrared temperature tester, and external microwave control box, as shown in Figure 5. The heating source used in the test was microwave source, the working power was 1.5 kW, and the working time could be flexibly controlled. In this paper, the heating time was set as 80 s. At present, microwave frequency of 2.45 GHz is mostly used in industrial microwave heating, while the working power of standard magnetron only reaches 1–1.5 kW. Therefore, in combination with engineering practice, a 2.45 GHz magnetron was used as the microwave heating source for microwave deicing. In addition, different temperature measuring points on concrete surface were selected to reflect the specific heating effect of the microwave source.

### 3.2. Test Method

(1) The microwave source port is controlled to be 20 mm above the concrete surface, the concrete specimen is heated by microwave for 80 s, and the surface temperature of each measuring point is recorded every 5 s.
(2) Simultaneously, the microwave source height of the device is adjusted to 20, 30, 50, 70, and 100 mm. The real-time temperature of each temperature measuring point is monitored and recorded every 5 s.
(3) Subsequently, the height of the microwave source is fixed as 20 mm. The surface temperature distribution of concrete specimen covered with ice is investigated and compared with that of a non-ice specimen.

## 4. Test Results and Analysis

Through tests, the specific data of concrete surface temperature distribution during microwave deicing and the influence of microwave source height and ice layer on it are obtained. By comparing and analyzing the data and integrating the changing trends, the following quantitative results can be obtained.

### 4.1. Variation Law of Concrete Surface Temperature During Microwave Heating

**Figure 6:**
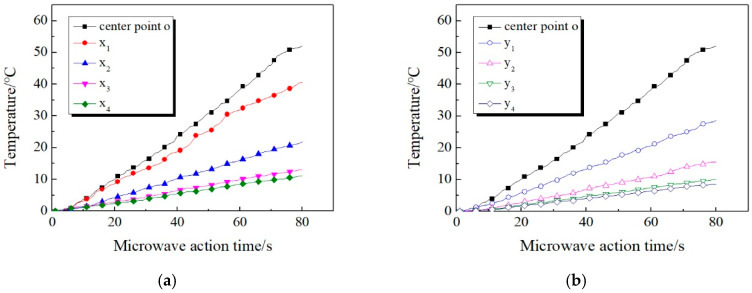
Temperature increase curve of measuring points. (**a**) *x*-axis; (**b**) *y*-axis.

The microwave source port is adjusted and fixed 20 mm above the concrete surface. Based on the experimental data, the real-time curves of temperature increase along the *x*-axis and *y*-axis are plotted respectively, as shown in Figure 6.

It can be clearly seen from Figure 6 that, in the overall trend, the temperature of every measuring point in the *x*-axis direction and *y*-axis direction exhibits a linear upward trend as the microwave action time increases. The R^2^ of the fitting line of each real-time temperature curve are 0.9964, 0.9922, 0.9967, 0.9951, 0.9961, 0.9980, 0.9979, 0.9955, 0.9967, respectively. R^2^ of each fitting line is above 0.99, indicating that the measured temperature increase curve is highly close to the straight line. It also reflects that when the height of the microwave source is constant, the electric field strength of the concrete surface is constant and does not change with time.

Through longitudinal comparison, it can be seen that the increase of the distance between the temperature measuring point and the center point will lead to the decrease of the temperature. Meanwhile, the temperature difference between two measuring points of same distance decreases as the distance between them and the center point increases. Among them, the center point temperature increases the most. When microwave is applied for 60 s, the temperature of the center point rises by 52.1 °C, which is higher than other measuring points in the *x*-axis direction and *y*-axis direction. With the increase of the distance between temperature measuring point and the center point, the temperature increase decreases gradually. For *x*_4_ and *y*_4_ which are farthest from the center point, the corresponding temperature increases are only 11 and 8.7 °C, which are 21.1% and 16.7% of the center point respectively. This indicates that when the distance between the specimen surface and the waveguide port is constant, the electric field strength on the concrete surface decreases significantly with the increase of the distance from the center point.

By comparing Figure 6a,b it can be seen that the temperature in the *x*-axis direction is always higher than that in the *y*-axis with the increase of the distance from the center point. This can be explained by the rectangular shape of the waveguide port. Although the temperature measuring points on the *x*-axis and *y*-axis directions are the same distance from the center point of concrete surface, the measuring points in the *x*-axis direction are closer to the projection position of the waveguide port, which results in a larger temperature increase.

In addition, the method of controlling variable is adopted to calculate and sort out the heating time of concrete surface under different microwave action times by taking 10 °C as the standard temperature value. Table 2 below can be obtained.

It can be seen that: (1) as the distance between the temperature measuring point and the center point of the concrete surface increases, the time for each temperature measuring point to reach the specified temperature increases significantly. For the center point o, only 20 s of microwave action time is required to reach 10 °C, while x_3_, which is only 45 mm from point o, takes 60 s to reach 10 °C. (2) The rise rate of edge temperature is very slow. The temperature of the measuring points has not reached the specified temperature within the microwave action time of 90 s. This indicates that the temperature increase rate on the concrete surface will affect the time for concrete to reach a certain temperature to a large extent.

**Figure 7:**
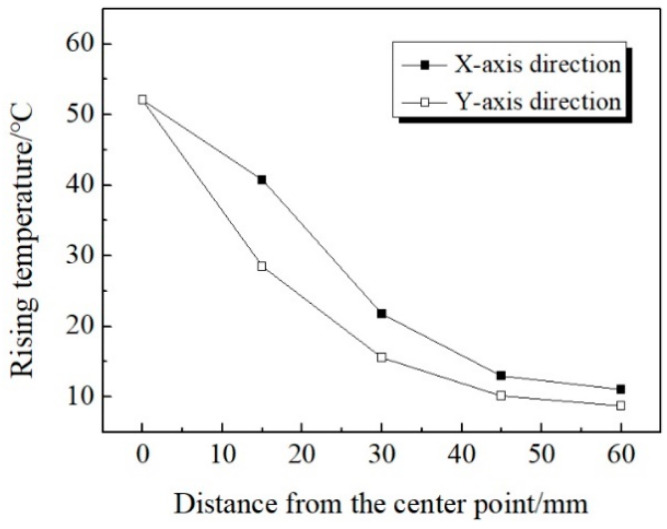
Rising temperature of measuring points.

By controlling the microwave action time to 75 s, temperature variation law of the measuring points on the *x*-axis and the *y*-axis can be further compared and analyzed. Based on this, the variation law in two directions can be obtained as shown in Figure 7.

It can be seen from Figure 7 that: (1) The temperature of the measuring point in the *x*-axis direction is always higher than that in the *y*-axis direction. (2) The rising temperature of *x*_1_ is 40.7 °C, while that of *y*_1_ is 28.5 °C, with a temperature difference of 12.2 °C, which is of significant difference. This can be explained by the fact that the waveguide port is rectangular and the microwave attenuation is faster on the shorter side. With the increase of the distance from the center, the temperature difference between the *x*-axis and *y*-axis decreases gradually. For the two temperature measuring points with same 60 mm distance from the center, the rising temperature of *x*_4_ is 11 °C and *y*_4_ is 8.7 °C, indicating that the temperature is very close. (3) As the distance from the center point increases, the decreasing degree of the rising temperature of the *x*-axis and *y*-axis temperature measuring points gradually slows down. For x_2_ with a distance of 30 mm from the center point, the rising temperature decreases by 30.3 °C, while for x_4_ with the same distance of 30 mm from *x*_2_, the rising temperature drops by 10.8 °C. 

### 4.2. Temperature Distribution of Concrete Surface at Different Microwave Source Heights

**Figure 8:**
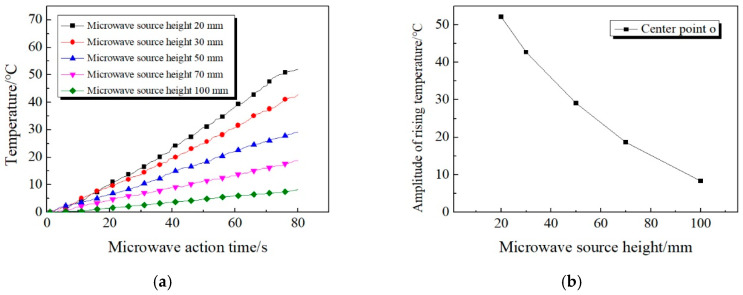
Temperature curve of the center point of concrete surface. (**a**) Real-time variation of temperature. (**b**) Amplitude of rising temperature.

By monitoring and analyzing the concrete surface temperature at different microwave source heights, the temperature change curve can be drawn, as shown in Figure 8 below.

It can be seen from Figure 8 that: (1) The amplitude of rising temperature decreases rapidly with the increase of microwave source height. When the height of microwave source is adjusted to 20 mm, the rising temperature at point o is 52.1 °C. However, when height is raised to 100 mm, the rising temperature is only 8.3 °C, which decreased by 43.8 °C. (2) As the height of the microwave source increases, the amplitude of the decrease in rising temperature reduce gradually. When the height increased from 20 to 50 mm, the rising temperature at point o decreased by 23 °C. However, when the height increased from 70 to 100 mm, the rising temperature only decreased by 10.4 °C.

**Figure 9:**
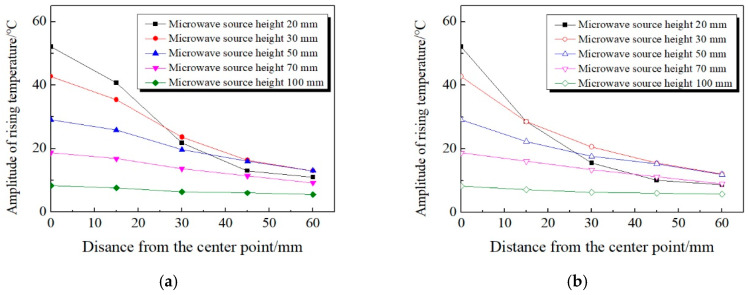
Variation rule of concrete surface temperature with microwave source height. (**a**) *x*-axis; (**b**) *y*-axis.

In addition, it can be concluded from Figure 8 that the temperature rising trend of the concrete surface presents different amplitude in the case of different waveguide heights. Therefore, taking the rising temperature of each point as index, the influence of the waveguide height on it has been drawn, as shown in Figure 9.

From Figure 9, it can be concluded that: (1) At different microwave source heights, the rising temperature of the center point of is always the largest relative to other temperature measuring points. As the distance between the temperature measuring points and center point o increases, the rising temperature of each measuring point decreases gradually. (2) The higher the microwave source height, the more uniform the temperature distribution of the concrete surface. When the height of the microwave source is adjusted to 20 mm, the rising temperature of center point o differs 51.8 °C from that of x_4_ point. However, when the height of the microwave source is adjusted to 100 mm, the difference is only 1.2 °C, which means that the surface temperature of the concrete is basically at the same level. (3) As the height of the microwave source increases, the temperature of each measuring point shows a downward trend. However, when the height is adjusted to 20 mm, the temperature of *x*_3_ and *x*_4_ is 9.6 and 6.6 °C respectively, which is lower than the corresponding rising temperature when the height is adjusted to 30 mm or even 50 mm. The reason for this phenomenon is that when the microwave source is 20 mm high, the temperature distribution of concrete surface is of great difference from that at other heights. At a height of 20 mm, the rising temperature of *x*_2_~*x*_4_ drops sharply, and the decline is much more significant than the rising temperature of heights of 30 mm and 50 mm.

Therefore, it can be known that the microwave source height is an important factor affecting the temperature increase, which further affects the heating efficiency in the microwave deicing process. The heating efficiency shows an upward trend with the decrease of the height of the microwave source. According to comprehensive analysis, the microwave source height of 20–30 mm reaches the optimal state of microwave deicing effect.

### 4.3. Effect of Ice Layer on Temperature Distribution of Concrete Surface

**Figure 10:**
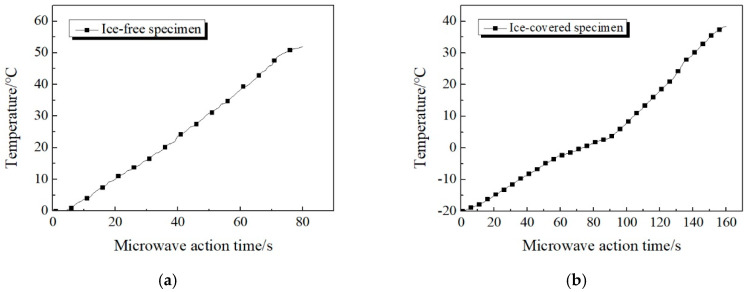
Temperature variation of two kinds of concrete specimen surfaces. (**a**) Temperature increase of ice-free specimen. (**b**) Temperature increase of ice-covered specimen.

Since the center point of the concrete surface is closest to the microwave source, the temperature change of the center point is taken as a reference. The height of the microwave source is controlled to 20 mm and the temperature distribution of the concrete surface covered with and without an ice layer is compared. A comparison chart of the rising temperature curve of the center point of the specimen is shown in Figure 10.

It can be seen from Figure 10 that the rising trend of temperature at the center point of concrete without ice cover is not entirely consistent with that under ice cover. The former shows a linear trend of temperature increase during the duration of microwave, while the temperature increase at the center of concrete surface covered by ice is not completely linear, which can be roughly divided into three stages. (1) The first stage is when microwave starts to work until the temperature of the measuring point is below 0 °C. At this stage, the temperature increase shows a linear trend. During the process, the microwave directly penetrates the ice layer to heat the concrete surface, and the concrete surface generates thermal effect under the microwave field. Since the ice layer is still in a negative temperature state, part of the heat absorbed by the concrete will also be transferred to the ice layer by heat conduction [24,25]. Thus, the temperature increase rate is not high. (2) When the surface temperature increases to 0 °C, it enters the second stage. The slope of the temperature increase curve has a certain decline, and the temperature change is not significant. This is because when the temperature is around 0 °C, the ice layer combined with the concrete surface begins to melt, which absorbs plenty of heat. At this stage, most of the heat absorbed by the concrete surface in the microwave field is used to heat and melt the ice layer, which leads to a significant decrease in the rate of temperature increase at the center measuring point. (3) The third stage is when the temperature of the center point is higher than 0 °C. At the beginning of this stage, the ice layer adhering to the concrete surface has basically melted into water, enabling microwaves to pass through the overlying ice and act directly on the water. Since water is a strong absorber of microwaves, the microwave energy it absorbs can release a large amount of heat, which causes the surface temperature of the concrete to rise rapidly. In this process, both concrete and water absorb microwave energy and then convert it into heat energy, which is more efficient and greatly improves the rate of temperature increase.

When the microwave source is 20 mm high, the rate of temperature increase at the center point is 0.293 °C/s in the first stage, 0.154 °C/s in the second stage, and 0.538 °C/s in the third stage. By comparison, the temperature increase rates of these three stages are successively from high to low: the stage above 0 °C, the stage below 0 °C, and the stage near 0 °C.

**Figure 11:**
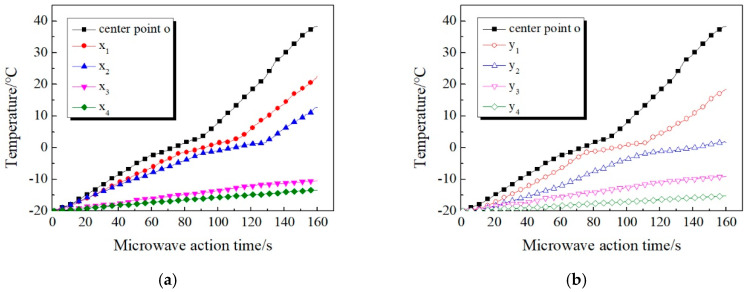
Temperature increase of ice-covered specimen surface. (**a**) *x*-axis (**b**) *y*-axis.

Figure 11 shows the comparison of temperature increase curves of ice-covered specimen in the *x*-axis and *y*-axis directions. It can be seen that: (1) The temperature measuring points at different positions of specimen all appear the above three stages during the heating process. However, the time at which each temperature measuring point enters the second stage is different, and the duration of this stage is different as well. (2) Within 160 s of microwave source operation, the temperatures of *x*_3_, *x*_4_, *y*_3_, and *y*_4_, which are far away from point o, are still below 0 °C. The bonding position between ice layer and concrete surface is still in freezing state and does not begin to melt.

It can be found from the above temperature variation law that the ice layer will affect the microwave heating efficiency, and thus affect the temperature distribution of the concrete surface during the microwave deicing process. The specific reason can be explained as that water molecules in the ice layer are frozen and do not rotate in the microwave field when heated by microwave. Therefore, the relative dielectric constant of ice is only 0.0009, which means the ability to absorb microwaves is extremely weak. When microwaves penetrate the ice layer, there is substantially no thermal effect [26]. In the practical deicing process, due to the tight combination of the ice layer and the road surface, the direct heating area of is the junction of both. Therefore, the low temperature of ice will inevitably slow down the heating rate of pavement materials. At the same time, the melting of ice into water will absorb plenty of heat from the surface material, which will also further reduce the heating rate.

## 5. Effect Mechanism of Microwave on Temperature Distribution of Concrete Surface

During the test, it is found that the microwave effect of concrete will disappear immediately after the microwave emission device stops. However, the surface temperature of the concrete specimen will still increase to a certain extent, thereby further melting the ice layer at the joints, which is called the residual heat effect. According to this phenomenon, tests have been carried out, and a real-time temperature curve of each measuring point within 20 min after microwave action stopped has been analyzed.

**Figure 12:**
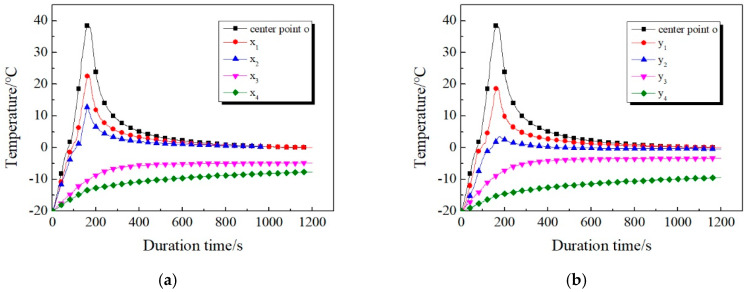
Real-time temperature of the whole process of the ice-covered specimen surface. (**a**) *x*-axis; (**b**) *y*-axis.

When the height of the microwave source is adjusted to 20 mm, the temperature variation of the ice-covered concrete specimen in the whole process is shown in Figure 12 above, including the microwave heating process and after stopping heating for 20 min. It can be seen that: (1) the temperature variation trend in the central area of concrete is the same as that without ice cover. In addition, the temperature of o point and the temperature measuring points nearby dropped rapidly after the termination of microwave action. (2) The temperatures of *x*_3_, *x*_4_, *y*_3_, *y*_4_, which are far away from the center point, have been rising slowly within 20 min after the termination of microwave action, and gradually approach 0 °C. For the concrete covered with ice layer, the concrete in the center area and the water melted from the joint have higher temperature after microwave irradiation. Because of the large specific heat capacity of water, a large amount of heat will be released when the temperature decreases. Some of this heat will continue to melt the remaining ice, while others will increase the temperature of the area farther from the center. (3) For the area with heating temperature higher than 0 °C, the temperature gradually tends to 0 °C due to continuous heat exchange with the low-temperature interface. Moreover, as a result of the large specific heat capacity of water and more heat storage, water will not solidify into ice in a short time.

It can be seen that the residual heat effect will affect the temperature distribution of the concrete surface, thus promoting the microwave deicing efficiency. Through the analysis of this phenomenon, the influence mechanism can be roughly summarized as follows: The residual heat effect can be analyzed separately, which consists of two sub-effects, namely melting effect and crushing effect. Among them, melting effect plays a leading role, and crushing effect is a staged embodiment of melting effect. The two sub-effects coexist and promote each other. Microwave generates thermal effects between concrete surface and ice layer. At the same time, due to the high specific heat capacity of concrete, heat produces a certain accumulation effect. During the deicing process, the ice layer begins to melt from the contact part with the concrete surface, creating a closed space between the two. With the end of microwave deicing, the concrete surface begins to release the heat accumulated in the previous period, which causes the temperature in the space to increase in a short time so as to further melt the ice layer. In addition, the heat generated by the microwave also cause a melting effect inside the ice layer, thereby forming a large number of small closed cavities inside. After the microwave action is over, the remaining microwaves will be reflected continuously inside these closed cavities, thus generating heat. Meanwhile, according to the theory of molecular kinematics, water molecules in the frozen state on the inner surface of the cavity also generate heat under the driving of remaining microwaves, which further promotes the melting effect. This phenomenon can be defined as a continuity of the melting effect. The other sub-effect is the crushing effect. After the microwave action is completed, not only the temperature distribution between concrete surface and ice layer changes, but also its physical properties change to a certain extent. As can be seen from the above, this characteristic change is mainly concentrated at the junction and the closed cavity inside the ice layer. When the cavity is formed, the ice melts into water and the density increases, which leads to the low density of the air inside the cavity, almost approaching the vacuum state. Based on this, a pressure difference is formed inside and outside the cavity. The air pressure directly crushes the ice layer and produces a crushing effect. Under the combined effect of the above two sub-effects, the temperature distribution of the concrete surface changes, resulting in a residual heat effect. It is also confirmed from the side that the premise of the temperature increase process in Figure 12 is faster, while the latter stage is relatively stable. The reason is mainly due to the leading role of the pre-melting effect. With the continuous disappearance of remaining microwaves and the gradual weakening of the crushing effect accompanying the entire process, the temperature also tends to be stable. For the above mechanism analysis, this paper conducts further verification through simulation and comparison experiments. For analysis of the above mechanism, further verification is carried out through simulation and comparison tests.

**Figure 13:**
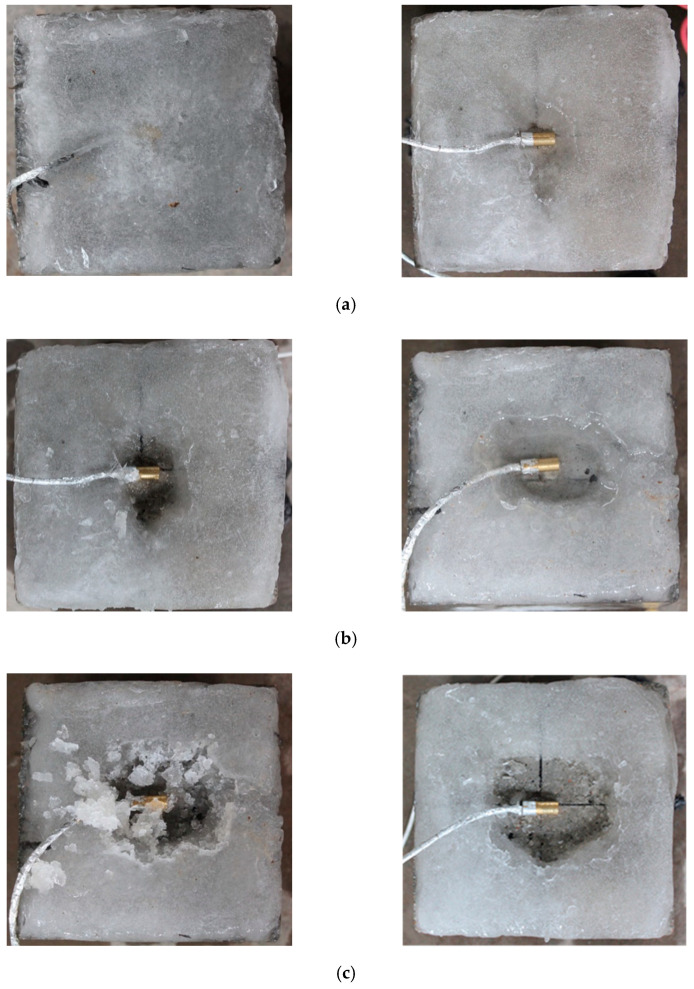
Ice state and breaking effect under different microwave action time. (**a**) 70 s; (**b**) 90 s; (**c**) 120 s.

During the test, the attached ice layer was knocked with a blade to simulate the mechanical deicing in reality. Figure 13 shows the ice state after treatment under different microwave action time. It can be seen that: (1) When the microwave action time in the early stage was less than 70 s, the ice attached to the concrete specimen was always frozen, with almost no change in the physical state. After the microwave effect stopped for 2 min, the specimen presented a state on the right side due to the residual heat effect. The ice around the center point began to melt, and the temperature sensor was partially exposed. By striking the ice with a shovel, it could be found that it was difficult to break through the ice due to the small broken part and larger surrounding frozen area. (2) When the microwave duration increased to 90 s, the ice melting range in the central region increased and the ice thickness above the melting region became thinner. Through the comparison of the left and right figures, it could be clearly seen that the residual heat effect promoted further melting of the ice layer, thereby improving the deicing efficiency. By crushing the ice with the same force, it could be observed that the melting area was easily broken, and the surface of the concrete specimen was relatively clean without residual ice. (3) When the microwave action time continued to increase to 120 s, the ice layer above the concrete center point had completely melted. During the subsequent process of ice removal, it was found that the strength of the ice layer had been basically lost, and the ice could be removed by tapping gently.

It can be seen from the above test phenomena that: (1) With the increase of microwave action time, the bonding part between ice layer and concrete surface gradually melts, forming the weak link of ice layer. (2) In the process of deicing, the higher the ice layer temperature is, the easier it is to remove. When the temperature is lower than 0 °C, the ice layer is difficult to remove and cannot be completely cleaned. (3) When the concrete surface temperature is higher than 0 °C, the ice layer at the joint has melted. At this point, knocking on the ice layer will cause the ice layer to rupture and be completely removed without residual ice. In summary, the above comparative simulation tests can effectively verify the validity of the theory mentioned above, which largely conforms to the experimental facts.

## 6. Conclusions

In recent years, microwave deicing technology is widely used in deicing of highway and airport pavement. Aiming at two key characteristics of microwave deicing process, namely surface temperature distribution law and influencing factors of pavement concrete, the real-time temperature change of concrete in microwave field, amplitude of rising temperature, ice layer breaking form, residual heat phenomenon, etc. are systematically studied in this paper. The main conclusions are as follows.

(1) In the process of microwave deicing, the microwave effect directly leads to the linear rise of the surface distribution of pavement concrete, among which the temperature at the surface center point is the most obvious. The increasing amplitude of concrete surface temperature is centered on the center point and shows a stepwise decreasing trend along the radius.
(2) The microwave thermal effect on concrete surface decreases with the increase of the microwave source height, but the uniformity of temperature distribution increases. When the height of microwave source is 20 mm, the irradiation blind area is generated, and the irradiation area is small, which reduces the effective range of microwave.
(3) The ice coverage will directly affect the real-time temperature curve of concrete surface under the action of microwave. In the case of no ice layer coverage, the real-time temperature curve increases linearly, and the microwave effect on the concrete surface decreases with the increase of the distance between the temperature measuring point and the center point. While in the case of ice cover, the temperature increase can be divided into three stages with 0 °C as the reference point, all of which show an upward trend. The heating rate is in order from large to small: the stage above 0 °C, the stage below 0 °C, and the stage near 0 °C.
(4) After the test, it is found that the surface temperature of airport pavement concrete can still be maintained above 0 °C after microwave deicing, and the retention time is 10 min. This phenomenon is defined as residual heat in the microwave deicing process, which can effectively delay the process of ice layer refreezing on the concrete surface.

Based on the above conclusions, this paper provides relevant theoretical basis and technical support for the microwave deicing technology of airport pavement, which has certain significance and promotion value. In future research, we will further study the microscopic mechanism and propagation mode of microwaves on the bonding surface of concrete and ice layer.

## Figures and Tables

**Table 1:** Concrete reference mix ratio.

**Material Consumption Per m^3^ of Concrete (kg)**	**Cement**	**Water**	**Large Stone**	**Medium Stone**	**Small Stone**	**Sand**	**Water-Reducing Agent**
	330	135	575	575	287.5	562.5	3.63

**Table 2:** Temperature increase time of each measuring point.

Measuring Point	o	*x*_1_	*x*_2_	*x*_3_	*x*_4_	*y*_1_	*y*_2_	*y*_3_	*y*_4_
Temperature increase time (s)	20	22	39	/	/	31	55	/	/
